# The Musculoskeletal 30-question multiple choice questionnaire (MSK-30): a new assessing tool of musculoskeletal competence in a sample of Italian physiotherapists

**DOI:** 10.1186/s12891-024-07400-6

**Published:** 2024-04-04

**Authors:** Giuseppe Giovannico, Marco Pappaccogli, Matteo Cioeta, Leonardo Pellicciari, Saad Youssef, Domenico Angilecchia, Gabriele Giannotta, Fabrizio Brindisino

**Affiliations:** 1 Department of Medicine and Health Science Vincenzo Tiberio, University of Molise c/o Cardarelli Hospital, C/da Tappino, 86100 Campobasso, Italy; 2 IRCCS San Raffaele Roma, 00166 Rome, Italy; 3 IRCCS Istituto delle Scienze Neurologiche di Bologna, Bologna, Italy; 4 Rehabilitation service - ASL Bari, Bari, Italy; 5 Scientific Institute IRCCS “E. Medea” - Unit for Severe disabilities in developmental age and young adults Developmental Neurology and Neurorehabilitation, Brindisi, Italy

**Keywords:** Surveys and questionnaires, Musculoskeletal diseases, Physical therapists, Professional competence, Consultation and referral

## Abstract

**Background:**

The prevalence and cost of musculoskeletal diseases increased dramatically over the past few decades. Therefore, several institutions have begun to re-evaluate the quality of their musculoskeletal educational paths. However, current standardized questionnaires inadequately assess musculoskeletal knowledge, and other musculoskeletal-specific exams have limitations in implementation. The musculoskeletal 30-question multiple choice questionnaire (MSK-30) was proposed as a new tool for assessing basic musculoskeletal knowledge***.***

**Aim:**

To analyse basic musculoskeletal knowledge in a sample of Italian physiotherapists by administering the MSK-30 questionnaire.

**Methods:**

After a transcultural adaptation process, the MSK-30 was developed and administered to Italian physiotherapists to assess their musculoskeletal knowledge. Participants were invited to participate in the survey via the SurveyMonkey link. Mann-Whitney test and the Kruskal-Wallis test with Bonferroni correction were used to observe the differences between groups in the MSK-30 scores.

**Results:**

Four hundred-fourteen (*n*=414) physiotherapists participated in the survey. The median MSK-30 value was higher in physiotherapists who attended the International Federation of Orthopaedic Manipulative Physical Therapists postgraduate certification than in those who attended unstructured postgraduate training in musculoskeletal condition or in those who had not completed any postgraduate training in this field (*p*<0.001).

**Conclusions:**

This work demonstrates significant differences in the management of musculoskeletal disorders between those with specific postgraduate university education and those without. The findings can contribute to the advancement of the physiotherapy profession in Italy. Authors recommend further research with more robust methodologies to deeper understand this topic. Musculoskeletal conditions will continue to represent a significant portion of primary care visits, and future generations of physiotherapists must be prepared to address this challenge.

**Supplementary Information:**

The online version contains supplementary material available at 10.1186/s12891-024-07400-6.

## Introduction

Musculoskeletal Conditions (MsC) are a significant contributor to global disability [1]. These disorders have a high prevalence throughout all ages, with at least one person out of five (children included) complaining of musculoskeletal pain, as seenable based on previous articles [2–4]. MsC contains more than 150 diagnoses affecting the musculoskeletal system [3]. The impact of these conditions is expected to increase with the global aging of the population, driven by age-related risk factors [4]. This trend will result in a rise in healthcare costs, which are already heavily influenced by MsC [5].

Taking into account all these factors, many institutions re-evaluated the significance of developing specific clinical competencies on this topic [6]. To establish if medical students were adequately experienced to face this rising problem, members of the University of Pennsylvania Orthopaedic Surgery Department developed a base competence questionnaire that could objectively evaluate the musculoskeletal knowledge of the health professional [7]. Usually known as the "Basic Competency Examination in Musculoskeletal Medicine" (BCEMM), this questionnaire has been repeatedly used in several American and European Universities to appreciate the adequacy of Medical didactic programs [8–10].

In 2019, the musculoskeletal 30-question multiple-choice questionnaire (MSK-30) was presented by Cummings et al. [11]. It is a helpful, multiple-choice questionnaire about the musculoskeletal competence evaluating the musculoskeletal competence of the healthcare professionals working in primary-care process. This questionnaire aimes instead to identify common and critical MsC, to choose appropriate initial management, and to know when to refer the patient to a professionalist. Furthermore, the exam format reduces the likelihood of misinterpretation compared to the short-answer format used in the BCEMM, thus allowing for more accurate statistical analysis [11]. By assessing individual musculoskeletal knowledge, this clinical evaluation tool identify weaknesses and address knowledge gaps. The accompanying answer explanation guide and included references further support this process.

Nowadays, direct access to physiotherapy care is a largely used method for managing musculoskeletal pain in many patients [12–14]. This trend needs advanced skills developed by the physiotherapists working in direct access to allow the best management of the patient's condition, including his referral, if necessary [15].

For these reasons, the aims of this study were to adapt the MSK-30 questionnaire into Italian language and to evaluate the basic musculoskeletal knowledge in a sample of Italian physiotherapists by administrating the Italian version of the MSK-30 questionnaire. The secondary aim of this work is to spark a deeper analysis on the necessity of carving university physiotherapy programs corporating these key topics.

## Methods

### Study design and ethical approval

This research project is an observational study of prevalence, conducted according to the CHERRIES Guidelines [16] and reported following the STROBE (Strengthening the Reporting of Observational Studies in Epidemiology) checklist [17].

The Ethical Committee of the University of Molise, Campobasso, Italy, approved the study protocol (Prot. n. 03/2021).

### Questionnaire development and pretesting

To adapt the questionnaire to the Italian context, a transcultural adaptation process of the Cummings' version was performed following the international guidelines [18] (the detailed steps followed for the transcultural adaptation of the MSK-30 into Italian language are shown in Appendix 1). The entire MSK-30 Italian version is reported in Appendix 2.

### MSK-30 implementation

The original questionnaire comprises 30 items developed and based on most common critical arguments of musculoskeletal medicine (i.e., trauma, infection, pediatrics, overuse, injuries, osteoarthritis, rheumatologic disease, environmental injury, head injury, and low back pain) [11]. The questions aimed to correctly identify MsC and the correct initial management, including the possibility of a referral to another health professional.

MSK-30 consists of 30 multiple-choice questions; each question has four alternative answers with only one correct option. Following the procedures described in the original article, the final score count assigned one point for every correct answer and was obtained by summarising every correct question. The final score was be expressed in percentage. There was no established minimum score for the test threshold.

Socio-demographic variables were implemented from Cummings study [11] and were investigated by 12 multiple-choice questions (i.e., age, sex, education, work ages). For the clinical questions, the authors decided not to modify the original ones as they reputed them to be complete and exhaustive in the topic investigation.

### Procedures

The survey was administered to Italian physiotherapists working in Italy during the survey sharing period; the participation was voluntary and anonymous.

The "Associazione Italiana di Fisioterapia" (AIFI) and the "Gruppo di Terapia Manuale e Fisioterapia Muscoloscheletrica e Reumatologica" (GTM) promoted the survey. These scientific associations emailed the survey to their associates, inviting them to complete the form. The survey was also promoted on the main Italian social network of physiotherapists identified by the authors. The survey was sent together with a presentation letter to the participants explaining the aim of the study. The presentation letter contained a terms of agreement button and explained that participation without reimbursement or payment was voluntary and anonymous.

To make a competencies comparison based on the Italian university system, the physiotherapists' samples were divided into education levels: three-year Bachelor Degree (BSc), Bachelor Degree with a Postgraduate Degree in Orthopaedic Manual Physical Therapy (OMPT), and Master of Science Degree (MSc). The qualification in OMPT is a specialized area of physiotherapy for managing musculoskeletal conditions. OMPT is recognized in Italy as the academic postgraduate degree program organised as Manual Therapy course, complying with the standards set by the International Federation of Orthopaedic Manipulative Physical Therapists (IFOMPT), a member organization of World.

### Inclusion and exclusion criteria

Participants were included if they were physiotherapists working on the Italian territory possessed a BSc in Physiotherapy (Class L/SNT2) or equivalent degree, were regularly enrolled in the Professional Register in the year of compilation of the survey and own a valid personal email address. Participants were excluded if they knew the questionnaire, had no knowledge of the Italian language, or had pending legal cases.

### Questionnaire administration

The questionnaire was spread as a survey through the "Survey Monkey" online platform (SurveyMonkey, Palo Alto, CA) [19]. The link to the survey was shared on social media platforms such as Facebook and Twitter, as well as instant messaging applications like WhatsApp and Telegram, or sent via email. Participation to the survey was voluntary and completely free of charge for the participants, and the questionnaire was completed anonymously. The first server interface consisted of an information sheet regarding the aim of the study.

All the interviewees completed a written informed consent form before participating. All study-related procedures were performed according to the principles of the statement of Helsinki [20]. The participation in the survey explicitly authorized the treatment of data and finalized the study development. The server was programmed to block access from the same IP address after receiving a complete and successful submission to prevent more than one compilation from the same user. Moreover, to prevent the same user from filling out the questionnaire using different IPs, respondents were asked to indicate own registration number with the Order of Physiotherapists they belong to. The respondent could edit every answer to the questionnaire during the compilation and navigate the survey by clicking the dedicated buttons to modify previous answers. However, after completing the survey, no further changes have been made by the user.

### Data collection

The survey administration period was limited to three months, between October 1^st^ and 31^st^ of December 2021. After three weeks of no responses, we decided to close the survey.

To protect the anonymity of the respondents, users' data was collected with hidden IP and registration number address of respondents and secured by a password. Once the surveys were compiled, the results were anonymously sent to the authors, that provided them to the blind statistical analyst for data analysis.

### Statistical analysis

Descriptive statistics were used to describe the characteristics of the sample. Moreover, to assess the groups' differences in the questionnaire score, the non-parametric Mann-Whitney test was used when the groups contained two categories (e.g., gender, IFOMPT postgraduate degree, model of care) and the Kruskal-Wallis test with Bonferroni corrections was run when the groups included more than two variables (i.e., age, origin, academic title, etc.).

All statistical analyses were performed using SPSS (version 25 for Windows; SPSS Inc., Chicago, IL; 2004), and the alpha value was set to *p*=0.05.

## Results

Four hundred and fourteen (*n*=414) Italian physiotherapists participated in this survey: 57.5% were males (*n*=238/414), and 42.5% were female (*n*=176/414). Average time for completion MSK-30 was 12 minutes. The detailed sample characteristics are reported in Table 1.

**Table 1:** Demographic characteristics of the sample (*N*=414)

**Variable**	**Frequency**	**Percentage**	**95% CI**
*Gender*			
Male	238	57.5	52.7 - 62.3
Female	176	42.5	37.8 - 47.3
*Age*			
<30 years	144	34.8	30.2 - 39.4
31-35 years	95	22.9	18.9 - 27.0
36-40 years	46	11.1	8.1 - 14.1
41-45 years	42	10.1	7.2 - 13.0
>45 years	87	21.0	17.1 - 24.9
*Region of origin*			
Northern Italy	214	51.7	46.9 - 56.5
Southern Italy	106	25.6	21.4 - 29.8
Central Italy	94	22.7	18.7 - 26.7
*Academic degree*			
Bachelor degree	262	63.3	58.6 - 67.9
Postgraduate degree	11	26.8	22.6 - 31.1
Master of Science degree	41	9.9	7.0 - 12.8
*Master IFOMPT*			
No	318	76.8	72.8 - 80.9
Yes	96	23.2	19.1 - 27.3
*Years of experience*			
<5 years	120	29.0	24.6 - 33.4
5-10 years	110	26.6	22.3 - 30.8
11-20 years	109	26.3	22.1 - 30.6
>20 years	75	18.1	14.4 - 21.8
*Working place*			
Private structure	215	51.9	47.1 - 56.7
Private structure affiliated with NHS	122	29.5	25.1 - 33.9
National Health Service	77	18.6	14.9 - 22.3
*Competence's areas*			
Musculoskeletal, Orthopaedic/Post-Surgery patients	311	75.1	71.0 - 79.3
Geriatric patients	42	10.1	7.2 - 13.0
Neurological patients	44	10.6	7.7 - 13.6
Others (cardiological, respiratory, paediatric)	17	4.1	2.2 - 6.0
*Model of care*			
Direct access	218	52.7	47.9 - 57.5
Patients sent by physiciand	196	47.3	42.5 - 52.2
*Postgraduate training in MsC*			
Yes, manual therapy courses (Maitland, Kaltenborn, others)	171	41.3	36.6 - 46.0
No	92	22.2	18.2 - 26.2
Yes, course exclusively addressed to the management of musculoskeletal disorders	91	22.0	18.0 - 26.0
Yes, University Master in Manual Therapy (IFOMPT)	60	14.5	11.1 - 17.9

*Abbreviations CI* confidence interval, *IFOMPT* International Federation of Orthopaedic Manipulative Physical Therapist, *NHS* National Health Service, *MsC* Musculoskeletal Conditions

The physiotherapists' samples were divided into three educational levels: three-year Bachelor Degree (BSc) (*n*=262), Bachelor Degree with a Postgraduate Degree in Orthopaedic Manual Physical Therapy (OMPT) (*n*=96), and Master of Science Degree (MSc) (*n*=41) aimed to make a competencies comparison based on the Italian University system. Moreover the sample were divided into who obtained an IFOMPT degree (*n*=96) and who did not (*n*=318), aimed to evaluate the quality of teaching in IFOMPT courses regarding screening for referral and red flag identification. The scores' differences between groups are shown in Table 2.

**Table 2:** Differences in MSK-30 score between groups

**Variable**	**Median** **(1**^**st**^**, 3**^**rd**^** quartile)**	***P* value**
*Age (years)*		
<30 years	18.0 (16.0, 19.0)	*p*=0.030** <30 years vs. >45 years: **p=0.046**
31-35 years	16.0 (14.0, 18.0)	
36-40 years	17.0 (15.0, 19.3)	
41-45 years	17.0 (14.8, 19.0)	
>45 years	16.0 (14.0, 19.0)	
*Region of origin*		
Northern Italy	17.0 (16.0, 19.0)	*p*=0.006** Northern Italy vs. Southern Italy: **p=0.008**
Central Italy	17.0 (14.8, 18.8)	
Southern Italy	16.0 (13.0, 19.0)	
*Academic degree*		
Bachelor Degree	17.0 (14.8, 18.3)	*p*<0.001** Master of Science vs. Postgraduate Degree: **p<0.001** Bachelor Degree vs. Postgraduate Degree: **p<0.001**
Master of Science Degree	16.0 (14.0, 18.0)	
Postgraduate Degree	19.0 (16.0, 21.0)	
*Master IFOMPT*		
Yes	19.0 (17.0, 22.0)	*p*<0.001*
No	16.5 (14.0, 18.0)	
*Years of experience*		
<5 years	18.0 (15.0, 19.0)	*p*=0.213**
5-10 years	17.0 (15.0, 20.0)	
11-20 years	17.0 (15.0, 19.0)	
>20 years	16.0 (14.0, 19.0)	
*Working place*		
National Health Service	16.0 (15.0, 18.0)	*p*<0.001** Private structure affiliated with NHS vs. Private structure: ***p*<0.001** National Health Service vs. private structure: **p=0.006**
Private structure affiliated with NHS	16.0 (15.0, 18.0)	
Private structure	18.0 (16.0, 20.0)	
*Competence's areas*		
Musculoskeletal, Orthopaedic, Post-Surgery patients	17.0 (15.0, 19.0)	*p*<0.001** Neurological patients vs. Musculoskeletal, Orthopaedic, Post-Surgery patients: ***p*=0.002** Geriatric patients vs. Musculoskeletal, Orthopaedic, Post-Surgery patients: ***p*=0.037**
Geriatric patients	16.5 (14.0, 18.0)	
Neurological patients	16.0 (14.0, 18.0)	
Others (cardiological, respiratory, paediatric)	17.0 (14.5, 18.5)	
*Model of care*		
Patients sent by physicians	17.0 (15.0, 18.8)	*p*=0.033*
Direct access	17.0 (15.0, 19.0)	
*Postgraduate training in MsC*		
No	16.0 (13.0, 18.0)	***p*<0.001** No vs. Yes, course exclusively addressed to the management of musculoskeletal disorders: ***p*<0.001** No vs. Yes, University Postgraduate Degree in Orthopaedic Manual Therapy (IFOMPT): ***p*<0.001** Yes, manual therapy courses (Maitland, Kalterborn, others) vs. Yes, course exclusively addressed to the management of musculoskeletal disorders: ***p*=0.015** Yes, manual therapy courses (Maitland, Kalterborn, others) vs. Yes, University Postgraduate Degree in Orthopaedic Manual Therapy (IFOMPT): ***p*<0.001**
Yes, course exclusively addressed to the management of musculoskeletal disorders	18.0 (16.0, 21.0)	
Yes, University Postgraduate Degree in Orthopaedic Manual Therapy (IFOMPT)	19.0 (17.0, 20.0)	
Yes, manual therapy courses (Maitland, Kalterborn, others)	17.0 (14.0, 19.0)	

*Abbreviations CI* confidence interval, *IFOMPT* International Federation of Orthopaedic Manipulative Physical Therapist, *NHS* National Health Service, *MsC* Musculoskeletal Conditions

Some demographic variables were analysed through non-parametric tests used for differences in groups. The dichotomous groups were analysed with the Mann-Whitney test, and those not dichotomous with the Kruskal-Wallis test

*P* < 0.05 values show differences between groups

Significant values are highlighted in bold

^*^Refers to the Mann-Whitney Test

^**^Refers to the Kruskal –Wallis Test

In the age subgroups, statistically significant differences in the MSK-30 scores were detected between those under-30 (*n*=144) and those over 45 (*n*=87), favouring under-30 respondents (18.0 vs. 16.0 points, *p*=0.046). Statistically significant differences in the MSK-30 scores were reported between those in Northern Italy (*n*=214) and those in Southern Italy (*n*=116), favouring Northern respondents (17.0 vs. 16.0 points, *p*=0.008). There is a statistically significant difference in the score between those who obtained a Master of Science Degree (*n*=41) and who received a Postgraduate Degree (*n*=11) (16.0 vs. 19.0 points, *p*<0.001) and between those who got a Bachelor Degree (*n*=262) and who obtained a Postgraduate Degree (*n*=11) (17.0 vs. 19.0 points, *p*<0.001).

Regarding the postgraduate specialization in MsC, statistically significant differences existed between those who obtained an IFOMPT degree (*n*=96) and who did not (*n*=318) (*p*<0.001).

For the clinical setting, physiotherapists working in private structure (*n*=215) report values with a median value of 18.0, two points higher than who works in the NHS (*n*= 77) (*p*=0.006) and who works in private structures affiliated with NHS (*n*=122) (*p*<0.001). Moreover, there was a statistically significant difference between those who practiced direct access (*n*=218) and who received patients sent by physicians (*n*=196) (*p*=0.033). Further detailed comparisons were resulted in Table 2.

## Discussion

The completed survey aligns with other surveys spread through the Italian physiotherapists to investigate their knowledge regarding different musculoskeletal topics [21–23]. The MSK-30 is the latest standardized exam created to evaluate the musculoskeletal knowledge of medical graduates and those involved in the primary care process [11]. Interestingly, unlike the work of Cummings et al. [11], we administered the questionnaire to physiotherapists and not to medical doctors.

The absence of a threshold for passing the examination does not allow estimating the minimum level of knowledge to ensure adequate competence in MsC. Analyzing the data in detail, some significant results can be noted. Firstly, physiotherapists with a Postgraduate Degree (19/30) scored significantly higher than those with a BSc (17/30) and MSc (16/30). The difference between postgraduate-qualified physiotherapists and those with an MSc suggests that the Postgraduate Degree may have greater clinical relevance for physiotherapists who pursue it after their Bachelor's degree.

This discrepancy may be because postgraduate training equips professionals with greater proficiency in gathering and critically analyzing the best available scientific evidence relevant to their field.

Therefore, introducing a basic approach to interpreting scientific literature during Bachelor's degree programs could be beneficial, potentially enhancing accessibility of such knowledge for physiotherapists.

Interestingly, no significant score differences were observed between physiotherapists with BSc and those with BSc and postgraduate training programs in MsC like Kaltenborn, Maitland, Mulligan, and others. This could be attributed to the predominantly practical and technical nature of these courses.

Equally interesting were the data regarding the age and working context of the sample. At the same time, there was no significant difference in the scores of physiotherapists with more experience compared to those with less working experience. However, a difference could be observed between those under 30 years of age (18/30 points) and those over 45 years of age (16/30 points). These seemingly conflicting findings could be justified by the profound legislative and academic change that began within the profession in the early 2000s and, therefore, by the profound differences that had characterized academic paths over the years, as further explained. Last but not least, it is important to consider the data relating to the working context: this result could be justified by the highly competitive nature of the private practice. Differential diagnosis skills appeared greater in physiotherapists who obtain specific training (in particular, an IFOMPT Postgraduate Degree). These results are in line with Giovannico et al. (2020) found in their study [15]: graduates and specialists in OMPT obtained higher scores than non-specialized colleagues. Additionally, manual therapy physiotherapists had higher different pass rates. They performed better than their non-specialist colleagues and even better than those specialized in Sports Physiotherapy.

Differential diagnosis is a major area of study emphasized in University Physiotherapy programs worldwide, representing a necessary skill for physiotherapists, especially in private clinical practice and direct access to primary care [24]. This work highlights how Italian physiotherapists have obtained medium-low success rates and it also points out the lack of attention given to screening for the most common MsC within Italian university programs. Considering the growing availability of direct access care for musculoskeletal patients in Italy, this necessitated the introduction of a significant amount of content pertaining to the screening of common MsC in physiotherapy degree courses. In Italy, the figure of the physiotherapist has undergone many changes in the last twenty years [25], transitioning from auxiliary figures to professional orders with ethical and judicial responsibilities [26, 27]. The legislative evolution of the figure of the physiotherapist requires greater attention on how future professional figures are being trained, especially in sensitive areas like musculoskeletal health. This field can significantly impact a state's Gross Domestic Product due to its substantial socioeconomic impact [28]. In this context, physiotherapists could play an essential role in reducing the burden on healthcare systems [29, 30]. Therefore, university courses for physiotherapy should be implemented, considering the evolving role of these professionals and equipping them to recognize pathological clinical characteristics of an extra-professional nature through appropriate screening processes [31]. The diagnostic suggestion of the physiotherapist should prioritize identifying red flags and, in the case of a positive systems analysis, guide an advisable referral to a specialist physician [32–35]. This highlights a potential inadequacy of the current duration of the physiotherapy degree. Aligning the program's content with the demands of the socio-cultural an healthcare system in which it operates and extending its duration, for instance, from the current three, to five years, could represent an evolutionary process. At the same time, Postgraduate Degrees, offered as a two-year specialization at the end of the five-years Bachelor Degree, could allows physiotherapists to acquire skills in a specific field (e.g. musculoskeletal, respiratory, cardiological). These changes in the university training of Italian physiotherapists could have an important clinical impact, as they would make treatments more appropriate and effective.

It is necessary to observe how there have already been considerable improvements regarding the differential diagnosis and the management of complex conditions by physiotherapists [36] starting from the birth of the Postgraduate OMPT programs, which have significantly increased professionals' skills in these areas. Numerous scientific articles authored by Italian physiotherapists who have undergone this training show their competence in managing a broader range of MsC [37–43]. This expanded skillset strengthens their ability to deliver comprehensive patient care within their scope of practice. Moreover, the MSK-30 evaluation tool could be a valuable instrument to assess the effectiveness of physiotherapy education in Italy. By applying it solely to physiotherapists trained through postgraduate OMPT programs, we could evaluate the long-term impact of this training on graduates' skills compared to those with a shorter training period. This would provide valuable insights into the quality of teaching and potential areas for curriculum development.

### Strengths and limitations of the study

The heterogeneity of the population investigated associated with the first administration of the MSK-30 questionnaire in Italian allows for the broadest vision of the objective of the study. Moreover, the questions' composition is perfectly suitable for teaching purposes as it provides the tested subject to easily trace the bibliographic reference in the literature for a direct study of the topic covered within the specification question. Finally, the ideal multiple-choice response mode compared to the open response test used in BCEMM [9] is helpful because it minimizes the impact of misinterpretation by allowing for better analysis.

However, careful analysis is necessary, and several limitations must be considered. First, due to the small sample recruited, it is impossible to generalize the entire population of Italian physiotherapists. Also, the form of administration, a survey, and the lack of time limit within which to complete the examination could lead participants to use external sources (any tool of a technical or paper nature or anything that can create an advantage over the knowledge of each participant) contaminating answers and data. Moreover, the previous administrations of MSK-30 took place in the presence and the mode of examination.

Finally, to reach the final version of the test, numerous healthcare workers were involved, almost all doctors and only one physiotherapist.

## Conclusion

This work aimed to investigate the skills of Italian physiotherapists regarding the management of musculoskeletal disorders, establishing that there are significant differences between those who hold a specific post-graduate university education and those who do not. It would be remarkable for this work to contribute to a significant improvement of the physiotherapy profession in Italy, supporting a process that has already accelerated in the past two decades.

The authors recommend further research, employing more robust methodologies, which has recently gained traction across several European countries and Universities. As MsC will continue to be a prominent concern in primary care, future generations of physiotherapists must be adequately equipped to address this growing challenge.

### Supplementary Information

**Supplementary Material 1.**

**Supplementary Material 2.**

## Data Availability

The datasets used and/or analysed during the current study are available from the corresponding author on reasonable request.
