# Supplementary material for: The Musculoskeletal 30-question multiple choice questionnaire (MSK-30): a new assessing tool of musculoskeletal competence in a sample of Italian physiotherapists
Source: BMC Musculoskelet Disord. 2024 Apr 4;25:265. doi: 10.1186/s12891-024-07400-6 (PMC10996259; doi:10.1186/s12891-024-07400-6)
Supplement: Supplementary file 1 — Supplementary Material 1. [file 12891_2024_7400_MOESM1_ESM.docx]

**APPENDIX 1.** Translation and Italian cross-cultural adaptation of the MSK-30

*Step 1: translation into Italian.* Two independent mother-tongue Italian translators developed two Italian versions of the MSK-30. One translator (GG) was a physiotherapist with 20 years of clinical experience and a lecturer in physiotherapist's University courses for physiotherapists. The other translator was a professional translator with no medical background.

*Step 2: synthesis.* The two independent translations were compared, and no discrepancies were noted. Both translators and an observer (MP) worked together on a final translation draft.

*Step 3: backward translation*. Starting from that draft, two English mother-tongue translators without medical backgrounds translated the questionnaire back into English.

*Step 4: expert committee.* The translators, a linguistic expert, and four physiotherapists (MP, GG, FB, MC) constituted a committee of experts. The linguistic expert was a MSc with 25 years of experience as an English teacher. The physiotherapists on the Committee of Experts had clinical expertise in back and neck diseases and had attended training courses in pathology screening for physiotherapists. The committee analysed the translated document and developed the pre-final Italian version of MSK-30 (MSK-30-I).

*Step 5: pretesting*. The MSK-30-I was administered to 30 subjects (30 physiotherapists and 30 physiotherapy students in one year of Manual Therapy specialization). Furthermore, a face-to-face interview between the respondents and the expert committee components was conducted to explore the possible difficulties encountered while filling in the questionnaire (e.g., unclear or confusing questions, unknown or uncommon words, and expressions) to ask about the experience in responding to the questions and to clarify further some of the answers given by the respondents.

In conclusion, the outcome of the pretesting stage was satisfactory, and it was determined that no changes to the questionnaire were necessary. After that, the final MSK-30-I was developed.
