# Supplementary material for: The Musculoskeletal 30-question multiple choice questionnaire (MSK-30): a new assessing tool of musculoskeletal competence in a sample of Italian physiotherapists
Source: BMC Musculoskelet Disord. 2024 Apr 4;25:265. doi: 10.1186/s12891-024-07400-6 (PMC10996259; doi:10.1186/s12891-024-07400-6)
Supplement: Supplementary file 2 — Supplementary Material 2. [file 12891_2024_7400_MOESM2_ESM.docx]

**APPENDIX 2** The MSK-30 paper-based survey

1) A 41-year-old woman presents with a 1-day history of a painful and swollen left elbow. She reports sustaining a puncture wound to her elbow several days prior but denies any major trauma. On exam, her elbow is erythematous and extremely tender to palpation with a deep puncture wound noted on lateral aspect of the

elbow. Active and passive range of motion is markedly limited by pain. Vitals signs notable for temperature of 100.4F (38C). What is the best next step in management?

A) Prescribe a course of antibiotics and follow up after completed

B) Treat empirically with colchicine and allopurinol

**C) Arthrocentesis**

D) Relative rest, ice, and NSAID’s

2) A 22-year-old male soccer player falls on an outstretched hand. He comes to clinic the next day complaining of wrist pain. On exam, he has tenderness over the anatomic snuffbox. X-rays of the wrist are negative. Of the

choices below, the best next step in management is:

A) Immediate referral to orthopedics for surgical management

B) Relative rest and NSAID’s for pain

**C) Short-arm thumb Spica cast and follow up in 2 weeks**

D) Long-arm cast for 6-8 weeks

3) A 17-year-old rugby player catches his ring finger on an opponent’s shorts and feels immediate pain. On

sideline examination, there is swelling of the distal ring finger. When the DIP joint is isolated, the patient is

unable to flex. Most appropriate management after the match is:

A) Buddy tape the ring finger to the long finger until symptoms resolve

B) Place in extension splint for 6 weeks

**C) Referral to orthopedics**

D) Relative rest for 2 weeks and re-evaluate

4) A 43-year-old male comes into clinic with worsening back pain for the last week. The pain is located in the

lumbar region and is noted to be severe in nature. On exam, there is no bony tenderness but there is decreased sensation on the medial aspect of the thighs bilaterally. Review of systems reveals overflow incontinence for 2 days. What is the most appropriate next step in management?

A) NSAID’s and follow up in 2 weeks

B) Physical therapy

C) Corticosteroid injection

**D) Urgent MRI**

5) A 16-year-old female distance runner comes into clinic with left-sided anterior knee pain. She says the pain

feels like it is beneath her knee-cap and is worse with going up and down stairs and running. What is the most

appropriate management of this condition?

**A) Addressing the underlying cause and targeted physical therapy**

B) Order MRI now

C) Referral to orthopedics for surgical management

D) Straight knee immobilizer for 2 weeks and gradual resumption of activity

6) An 18-year-old football player injured his foot and ankle after it was stepped on during a game. He is able to bear weight on the foot but has significant pain in the midfoot region. Which of the following findings on

history and physical exam would be an indication for x-rays?

A) Pain with weight-bearing on injured foot/ankle

**B) Tenderness on palpation of the 1st/2nd metatarsal bases**

C) Tenderness over the lateral foot distal to the fibula

D) Tenderness at the anterior aspect of the medial malleolus

7) A 28-year-old new mother presents with right sided wrist pain. She locates the pain to the distal aspect of her radius. On exam, forced ulnar deviation of the wrist with the thumb grasped in a fist reproduces the patient’s

pain. The most likely diagnosis is:

A) Arthritis of the 1st carpometacarpal (CMC) joint

B) Distal radioulnar dissociation

**C) De Quervain’s tenosynovitis**

D) Triangular fibrocartilage complex injury

8) A 17-year-old high school football player is tackled and lands directly on the point of his left shoulder, causing him immediate pain. He points to the superior aspect of his shoulder when asked to locate the pain. On exam, his pain is reproduced when he attempts to reach across his body with the affected arm. What is the most likely diagnosis?

A) Deltoid muscle tear

**B) Acromioclavicular joint sprain**

C) Rotator cuff tear

D) Labral tear

9) A 64-year-old male with past medical history of hypertension, hyperlipidemia, and prostate cancer comes to the clinic complaining of new onset back pain that has woken him up from sleep on multiple occasions. Physical exam is unremarkable. The best next step in management is:

A) Physical Therapy

B) NSAID’s

**C) Imaging of spine**

D) Rest and follow up in 2 weeks

10) An 18-month-old toddler is brought to the emergency department for irritability, fever of 101.5°F, and refusal to walk or bear weight. The infant refuses to move the right hip and cries with passive motion. Ultrasound of the hip shows fluid in the joint. Of the choices listed, what is the most likely diagnosis?

**A) Septic hip**

B) Transient synovitis

C) Legg-Calve-Perthes disease

D) Developmental dysplasia of the hip

11) A man collapsed near the finish line of a marathon and was brought to the race medical tent. On exam, he is confused and has hot, dry skin. His core temperature is 105°F, what is your next immediate step in

management?

A) Transfer to closest emergency department 30 minutes away

**B) Immediate cooling with best method available**

C) Administration of IV fluids

D) Rehydrate with oral fluids

12) A high school football player comes into clinic after injuring his knee when he was tackled during practice. On inspection of the knee, he has a moderate effusion. Examination of the knee is limited by guarding and he has pain with motion of the knee. Which of the following should be included high on the differential diagnosis?

A) ACL tear

B) Osteochondral lesion

C) Medial meniscus tear

**D) All of the above**

13) An athlete who suffered a concussion and is still symptomatic with some light sensitivity and mild exertional headache. He can be allowed to return to play if:

A) The athlete’s symptoms have been improving for at least 2 weeks

B) Imaging of the head is normal

C) The athlete has physically and mentally rested for at least 1 week

**D) The athlete should never return to play while symptomatic**

14) A 42-year-old woman comes into clinic with 3 weeks of right-sided anterolateral shoulder pain that is made worse when reaching overhead and laying on the affected side at night. Forward flexion of the shoulder to 90 degrees and forced internal rotation reproduces her pain. What is the initial step in management of this

condition?

A) Intraarticular corticosteroid injection

B) MRI for suspected rotator cuff tear

C) Arthroscopic subacromial decompression

**D) Activity modification and physical therapy**

15) A 31-year-old male comes into clinic with 2 weeks of lower back pain after helping a friend move into a new house. He describes the pain as dull and says it is diffuse but does not radiate down his leg. Physical exam

reveals tenderness to the paraspinal muscles in the lumbar region but is otherwise unremarkable. What is the

most appropriate next step in management?

A) X-rays of lumbar spine

B) Oxycodone

C) Referral for epidural steroid

D) **None of the above**

16) An 18-year-old female who runs cross country comes into clinic with 1 month of worsening right-sided deep groin pain. Pain is made worse with any weight-bearing activities. Review of systems is remarkable for

amenorrhea and a BMI of 19. What is the best next step?

A) **Imaging**

B) Physical therapy

C) NSAIDs

D) Intra-articular corticosteroid injection

17) Last night, a 25-year-old male sustained a displaced tibial fracture after a fall which was surgically repaired.

Today, he complains of increasing pain in his leg and some tingling in his toes. On physical exam, he is afebrile

and has a tense anterior compartment. When you consult the on call orthopaedic surgeon, what diagnosis are

you most concerned about?

A) Infection

B) Deep venous thrombosis

C) Redisplacement of the fracture

**D) Compartment syndrome**

18) A 15-year-old girl fell off her bike and landed on her shoulder. She complains of severe pain with movement of the shoulder. There is bruising and tenderness over the midpoint of the clavicle. X-ray shows a nondisplaced midshaft clavicle fracture. Which of the following is the most appropriate treatment for this patient?

**A) Sling for comfort and early range of motion as pain improves**

B) Referral to orthopedics for surgical repair

C) Shoulder immobilization for 4-6 weeks until evidence of fracture healing

D) Return to activity as tolerated without restrictions

19) A 21-year-old male presents with severe back and leg pain as well as dark urine after a strenuous weightlifting workout yesterday. His initial creatine kinase (CK) level is 17,523U/L (reference 25-90U/L). What is the next step in management for this patient?

**A) Aggressive hydration**

B) Muscle biopsy

C) NSAID’s and reassurance

D) Follow up CK measurement in 72 hours

20) A 45-year-old female who recently started playing tennis regularly comes into clinic with pain in her Achilles tendons bilaterally. Ultrasound confirms mid-substance Achilles tendinopathy bilaterally. The most

appropriate initial management of this condition is:

A) Referral to orthopedics for surgical management

B) Corticosteroid injection

**C) Rehabilitation focused on eccentric exercises**

D) Platelet-rich plasma injection

21) A 10-year-old basketball player comes in to clinic with gradual onset of left heel pain which is worse with

running and jumping. On examination, he has tightness of the gastroc-soleus complex and pain with the calcaneal squeeze test. What is the most likely diagnosis?

**A) Sever’s disease**

B) Calcaneal stress fracture

C) Achilles tendinopathy

D) Plantar fasciopathy

22) A 59-year-old woman comes in to clinic with 2 weeks of pain and numbness in her thumb, index finger, and long finger. She says the pain is worse at night and is relieved by shaking or flicking her wrist. Inspection of the hand reveals atrophy of the thenar eminence. What is the next best step in management?

A) Refer to occupational therapy

B) Corticosteroid Injection

C) Lifestyle modification

**D) Referral to orthopedics**

23) A 68-year-old male comes to the physician with worsening neck pain with radiation down his right arm,

consistent with cervical radiculopathy. Which of the following findings would necessitate immediate referral

to a spine surgeon?

A) Pain exacerbated by forced extension of the neck

**B) Hyperreflexia of the lower extremities**

C) Decreased sensation to light touch in the lateral arm

D) Pain isolated to the shoulder girdle

24) A 24-year-old male presents with several months of low back pain which lasts for 60 minutes in the morning and improves with activity but not with rest. Physical exam is remarkable for tenderness at the SI joints and positive FABER test. Review of systems is positive for increased fatigue lately. What x-ray finding is characteristic of this condition?

**A) Sclerosis of iliac side of sacroiliac joints**

B) Anterior displacement of L5 on S1

C) Excessive lumbar lordosis

D) Fracture of the pars interarticularis

25) A tall, lanky 13-year-old boy presents with vague left knee pain and a limp for one week, but a normal knee exam and pain with internal rotation of the hip. AP pelvis and frog-leg view of the L hip are shown below. What is the next step in management?


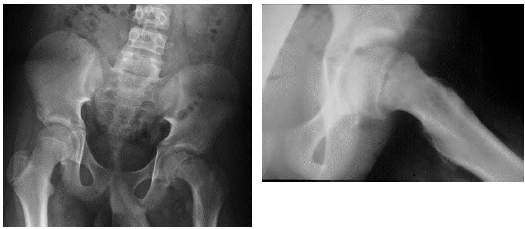


A) Allow to return to sports as tolerated

B) Refer for physical therapy and follow up in

4-6 weeks

C) Joint aspiration and synovial fluid analysis

**D) Immediately make non-weightbearing and refer to orthopedics**

26) A 12-year-old boy severely twists his ankle. Radiographs show only soft-tissue swelling. He is only tender at the distal aspect of the fibula. What diagnosis must be considered in addition to ligament sprain?

**A) Physeal injury**

B) Syndesmotic disruption

C) Peroneal tendon tear

D) Tarsal tunnel syndrome

27) During a physical altercation, a 21-year-old male sustains a 5th metacarpal neck fracture and a 3-mm wound proximal to the fracture. What is the next step in management?

A) Closed reduction and casting

B) Reduce and splint fracture plus oral antibiotics

**C) Irrigation and debridement of 5th MCP joint**

D) Open reduction and internal fixation of fracture

28) A 32-year-old male begins training for his first marathon and after 2 weeks experiences left-sided heel pain that is worse with his first few steps in the morning and gets better as the day goes on. Tenderness to what

area of the foot would most likely confirm your diagnosis?

**A) Medial calcaneal tubercle**

B) Achilles tendon insertion

C) Navicular tuberosity

D) Lateral calcaneal tuberosity

29) A 62-year-old female with hypertension, diabetes, and obesity comes into clinic with chronic left knee pain. Anterior-Posterior weight bearing radiograph of the knee shows medial joint space narrowing and osteophyte formation. Which of the following is the most appropriate initial management?

A) Referral to orthopedics for joint replacement

B) Obtain MRI of the knee

C) Limit weight bearing until pain resolves

**D) Recommend weight loss and exercise program**

30) Which of the following findings during a pre-participation physical for a 16-year-old male does not require

further investigation before clearing the individual to participate?

**A) A blood pressure of 138/89**

B) A systolic murmur that increases in intensity with the valsalva maneuver

C) History of a recent concussion without successful completion of return to play protocol

D) Family history of an uncle who died unexpectedly at age 26
